# The Tumor Immune Microenvironment in Breast Cancer Progression

**DOI:** 10.2340/1651-226X.2024.33008

**Published:** 2024-05-23

**Authors:** Marit Otterlei Fjørtoft, Kanutte Huse, Inga Hansine Rye

**Affiliations:** a Department of Cancer Genetics, Institute for Cancer Research, Division of Cancer Medicine, Oslo University Hospital, Radium Hospital, Oslo, Norway; b Institute of Clinical Medicine, University of Oslo, Oslo, Norway; c Department of Cancer Immunology, Institute for Cancer Research, Division of Cancer Medicine, Oslo University Hospital, Radium Hospital, Oslo, Norway; d Precision Immunotherapy Alliance, University of Oslo, Oslo, Norway

**Keywords:** Breast cancer, tumor immune microenvironment, subtypes, progression

## Abstract

**Background:**

The tumor microenvironment significantly influences breast cancer development, progression, and metastasis. Various immune cell populations, including T cells, B cells, NK cells, and myeloid cells exhibit diverse functions in different breast cancer subtypes, contributing to both anti-tumor and pro-tumor activities.

**Purpose:**

This review provides an overview of the predominant immune cell populations in breast cancer subtypes, elucidating their suppressive and prognostic effects. We aim to outline the role of the immune microenvironment from normal breast tissue to invasive cancer and distant metastasis.

**Methods:**

A comprehensive literature review was conducted to analyze the involvement of immune cells throughout breast cancer progression.

**Results:**

In breast cancer, tumors exhibit increased immune cell infiltration compared to normal tissue. Variations exist across subtypes, with higher levels observed in triple-negative and HER2^+^ tumors are linked to better survival. In contrast, ER^+^ tumors display lower immune infiltration, associated with poorer outcomes. Furthermore, metastatic sites commonly exhibit a more immunosuppressive microenvironment.

**Conclusion:**

Understanding the complex interaction between tumor and immune cells during breast cancer progression is essential for future research and the development of immune-based strategies. This comprehensive understanding may pave the way for more effective treatment approaches and improved patients outcomes.

## Introduction

The role of the immune system is to eliminate pathogens and aberrant cells through immune surveillance. However, this process becomes unsustainable as tumors gradually change the tumor immune microenvironment (TIME) into an immunosuppressive state, evading the host’s immune defenses. Tumors employ diverse strategies to escape immune detection, including secretion of immunosuppressive cytokines, downregulation of major histocompatibility complex (MHC) class I, and recruitment of tumor promoting immune cells [1]. The balance between pro- and anti-tumor immune cells emerges as a critical determinant influencing the progression of cancer.

The breast is not an immune-cell rich organ, and breast cancer has not traditionally been recognized as an immunogenic cancer. However, emerging evidence reveals varying degrees of immune cell infiltration across the different breast cancer subtypes. Triple negative breast cancer (TNBC), which lacks expression of human epidermal growth factor 2 (HER2) and the hormonal receptors estrogen and progesterone (ER and PR), and HER2^+^ breast cancer exhibit higher degree of immunogenicity compared to ER^+^ tumors. The degree of immune infiltration is hypothesized to reflect the tumor mutational burden, which is higher in TNBC and HER2^+^ tumors due to genomic instability, leading to increased neoantigen presentation [2].

An in-depth knowledge of the TIME is crucial for understanding tumor progression and in the development of novel targeted therapeutic strategies against breast cancer. In this review, we examine the composition of immune cells and their key roles in the molecular subtypes of breast cancer and through progression from normal breast tissue to metastatic disease.

## Immune microenvironment in normal breast tissue

The presence of immune cells in normal breast tissue is relatively scarce. Interestingly, higher immune infiltration is observed in healthy individuals with high risk of developing breast cancer, such as BRCA1 mutation carriers [3]. The immune microenvironment in breast tissue primarily consists of CD8^+^ T cells, CD68^+^ macrophages, and CD11^+^ dendritic cells (DCs) [4–7]. These immune cells are predominantly localized in the breast lobular and ductal regions, residing in close proximity to the epithelial cells [4–6]. The CD4^+^ T cells and CD20^+^ B cells are less frequent, and often completely absent from the breast [4]. Recently, a comprehensive study by Kumar et al. [7] using single cell RNA sequencing, identified CD8^+^ and CD4^+^ T cells and M1 macrophages to be the most prevalent immune cells. The CD8^+^ T cells expressed RUNX, indicative of a tissue-resident phenotype. B cells were found in lower numbers, and were dominated by Immunoglobulin G (IgG) and Immunoglobulin A (IgA) producing plasma cells [7]*.*

The breast is an organ undergoing constant change throughout life, influenced by hormonal fluctuations during puberty, the menstrual cycle, and pregnancy. The immune microenvironment is also altered by these hormonal fluctuations [8]. Additionally, age-related alterations are observed in the distribution and localization of immune cells, including decreased B and T cell density in peri-epithelial regions and increased M2 macrophages in the intralobular stroma [9]. These observations support the theory of immunosenescence during aging.

## Tumor immune microenvironment in breast cancer

**Figure 1:**
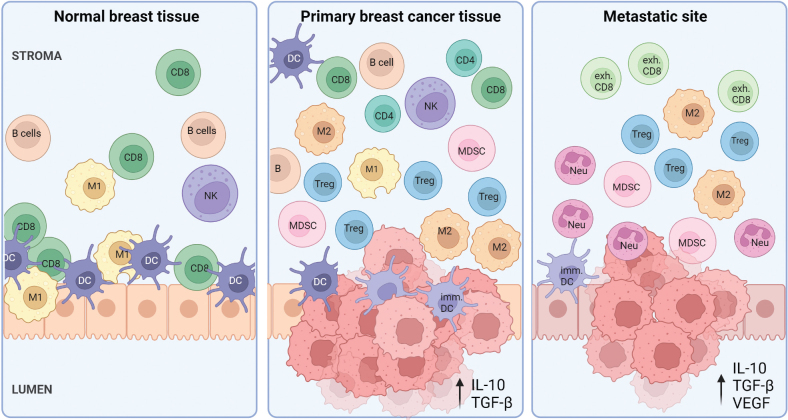
The variation in immune microenvironment from normal breast tissue through the immune escaping invasive cancer to distant metastatic sites. In normal breast tissue, immune cells are located most predominantly within the epithelial regions of the lobules, where CD8 T cells, DCs, NK cells and M1 macrophages are the most dominant cell types. In primary breast tumors, the amount of immune cells increases, where immunosuppressive cells such as Tregs, MDSCs and M2 TAMs aid tumor progression. The TIME in metastatic sites is highly immunosuppressive, including pro-tumor neutrophils, immature DCs and exhausted cytotoxic T cells. CD8=CD8^+^ T cell, exh.CD8=Exhausted CD8^+^ T; Treg=Regulatory T cell; DC=Dendritic cell; imm. DC=Immature DC; M1=M1 macrophage; M2=M2 macrophage; NK cell=Natural killer cell; MDSC=Myeloid-derived suppressor cell; Neu=Neutrophil. Created with BioRender.com.

In breast cancer, we see an increased presence of immune cells compared to normal breast tissue; this is summarized in Figure 1. Immune cells of both the lymphoid and myeloid lineage contribute to the dynamic changes seen during tumor progression (Table 1).

**Table 1:** Summary of immune cells, their main markers, and function in the tumor immune microenvironment in breast cancer.

Lineage	Cell type	Main markers	Functions
Lymphoid	B cells	CD19^+^	Recognize and present tumor antigens to activate T cells, produces tumor-specific antibodies, mediate proinflammatory signaling through the secretion of IFN-γ and direct killing of tumor cells by granzymes. (17–19)
	CD4^+^ T cells	CD3^+^ CD4^+^	Assist CD8^+^ T cells during the anti-tumor response through the secretion of various cytokines, activate B cells for antibody secretion, and activates macrophages to destroy ingested pathogens. (12,13)
	CD8^+^ T cells	CD3^+^ CD8^+^	Recognize and eliminate cells through the release of membranolytic proteins such as perforin and granzymes (14)
	Regulatory T cells (Tregs)	CD3^+^ CD4^+^ CD25^+^ FoxP3^+^	Specialized subset of CD4^+^ T cells involved in the regulation of T and B cell activation. Recruited to the TIME by chemokines and cytokines such as CXCL12, produced by tumor cells and other immunosuppressive cells. Can suppress host immune response by direct cell-cell contacts through CTLA-4 and LAG-3 inhibitory signals, and granzyme/perforin expression and production of immunosuppressive metabolites and cytokines e.g. (IL-10 and TGF-β). Can activate TGF-β secretion by tumor cells, a major cancer immune-escape mechanism. (15–17)
	Natural killer cells (NKs)	CD56^bright/dim^ CD16^+/-^	Monitor and kill abnormal cells. Have the unique ability to recognize and eliminate cells that lack expression of MCH class I, a common evasion strategy for tumor cells. Produce cytokines important for immune surveillance, such as IFN-γ and TNF-α. (18,19)
	Dendritic cells (DCs)	CD11c^+^ CD123^+^	Potent antigen presenting cell (APC), initiate adaptive immune responses by engulfing and presenting tumor-specific antigens on MCH class I and II molecules to T cells and producing immunomodulatory signals. Produce type 1 interferon that promote anti-viral and anti-tumor responses. (20,21)
Myeloid	M1 macrophages	HLA-DR^+^CD68^+^iNOS^+^	Eliminate pathogens and tumor cells through direct phagocytosis, activate T cells and NK cells through antigen presentation and secretion of proinflammatory cytokines and chemokines such as TNF-α, IL-1, and CXCL10. (22,23)
	M2 macrophages	CD68^+^CD163^+^	Secrete cytokines such as TGF-β and IL-10 which suppress cytotoxic CD8^+^ T cells and stimulate Tregs. Promote tumor cell proliferation, angiogenesis, and tissue remodeling through production of growth factors and chemokines such as EGF, FGF, VEGF, and TGF-β. (22,23)
	Myeloid-derived suppressor cells (MDSCs)	CD11b^+^ CD14^-/+^ CD15^-/+^ CD33^+^ HLA-DR^-/low^	Inhibit immune cells such as T cells, DCs, and NK cells, promote angiogenesis and tumor metastasis. Can induce severe anergy of effector immune cells, recruit Tregs at the tumor site, and drive the polarization of M2-like tumor-associated macrophages (TAMs). Inhibit antigen-specific T-cell tolerance, and suppress T-cell responses in an antigen- and neoantigen-specific manner. (6,15,24,25)

VEGF=vascular endothelial growth factor, IFN=interferon, IL=interleukine, TGF=tumor growth factor, TNF=tumor necrosis factor, CTLA-4=cytotoxic T-lymphocyte associated protein 4, LAG-3=lymphocytes activation gene 3

### Tumor infiltrating lymphocytes in breast cancer subtypes

Tumor infiltrating lymphocytes (TIL) have migrated from the blood stream to the tumor site. TILs encompass a large group of cells, including T cells, B cells, and NK cells. TILs are recognized for their anti-tumor properties, and it is well-established that high numbers of TILs are correlated with a beneficial prognosis in breast cancer [10–12]. High numbers of TILs are also associated with increased likelihood of response to neoadjuvant chemotherapy in all the molecular subtypes [13]. TILs can be classified as stromal (sTIL) or intratumoral (iTIL). Generally sTILs tend to be of higher prevalence than iTILs, and higher sTILs are associated with longer survival in all subtypes [13].

Within the TIL population, T cells with a memory phenotype emerge as the most abundant, playing a pivotal role in the immune response against tumors [14]. Specifically, CD8^+^ T cells serve as effector cells engaged in eradication of tumor cells through recognition of tumor-associated antigens and neoantigens presented by MHC class I. Simultaneously, CD4^+^ T cells provide support to CD8^+^ T cells by secreting a diverse range of effector cytokines.

B cells represent a minority among the TILs, yet their presence holds significance in relation to the formation of tertiary lymphoid structures (TLS). TLS are aggregates of lymphocytes in non-lymphoid tissue. In breast cancer these are found in the stroma and are associated with high-grade tumors [15]. In the context of triple negative breast cancer, these associations are particularly noteworthy, with TLS identified in higher abundance compared to HER2^+^ and ER^+^ subtypes [14]. Tumor infiltrating B cells are associated with an improved clinical outcome in breast cancer [16, 17], although their exact role in anti-tumor activity is not yet fully understood.

Regulatory T cells (Tregs) accumulate in breast cancer tissue compared to normal breast tissue [18], and infiltration of Tregs is correlated with high tumor grade, positive lymph node status and short overall and recurrence-free survival [19]. The prognostic role of Tregs in breast cancer is debated, and some studies have shown opposite results, as reviewed by Saleh and Elkord [19]. Thus, the prognostic effect of Tregs is dependent on the histological grade and molecular subtype.

Natural killer (NK) cells are important cytotoxic cells involved in immune surveillance and direct killing of aberrant cells [20, 21]. In breast cancer, estrogen is well known to have a suppressive effect on NK cells [22, 23]. The presence of NK cells is significantly associated with TILs and Ki-67 index [24]. Because of its killing functions NK cells can be useful in new forms of immunotherapy.

#### Triple negative breast cancer

Triple negative breast cancer has frequently high infiltration of TILs [25], predominantly CD8^+^ and CD4^+^ T cells. B cells [14] and NK cells [24] are also increased in TNBC compared to other subtypes, and the main B cell subpopulation in TNBC is memory B cells, with lower amounts of naïve B cells and plasma cells [26]. Tregs are predominantly found in immune infiltrated TNBC and ER^-^HER2^+^ subtypes [27, 28]. TNBC with elevated immune infiltration demonstrates enhanced survival rates and increased pathological complete response (pCR) during neoadjuvant therapy [29]. An increased presence of CD8^+^ T cells is reported to be associated with ER and PR negativity [28, 30], and has favorable prognostic value in ER^-^ tumors [31]. Surprisingly, while a robust presence of NK cells is associated with a favorable prognosis in ER^+^ and HER2^+^ breast cancer patients, a high presence in TNBC correlates with poor prognosis [32]. This can be explained by the dual role of NK cells as they can also exhibit pro-tumor functions. CD56^bright^CD16^dim^ NK cells in breast and colon cancers have been found to express the pro-angiogenic factor vascular endothelial growth factor (VEGF), which has a major role in tumor vessel growth and development of an immunosuppressive environment [33, 34]. In a suppressive TIME, NK cells can become dysfunctional due to molecular signals produced by tumor cells and environmental factors such as hypoxia and nutrient deprivation [35].

#### HER2^+^ breast cancer

HER2^+^ breast cancers are, alongside with TNBC, the subtypes with highest abundance of TILs [28]. The presence of TILs is associated with a favorable prognostic value in both ER^-^HER2^+^ and ER^+^HER2^+^ tumors [31]. Additionally, in HER2^+^ breast cancer treated with adjuvant chemotherapy, higher TIL abundance is associated with increased overall survival [13]. An increased presence of CD8^+^ T cells is associated with favorable prognosis in ER^-^HER2^+^ tumors [31]. Conversely, an increased presence of Tregs is associated with HER2 overexpression and decreased overall and progression-free survival [30]. In a spatial context, high CD8^+^ cell and Treg infiltration in the tumor bed is linked with a decreased survival, while a high CD8^+^ to Treg ratio in the surrounding area is associated with improved survival [30]. Interestingly, a strong presence of NK cells is associated with positive prognosis in patients with HER2^+^ subtype, opposite of what is seen in TNBC [24]. Deconvolution methods identified B cell IgG signatures as more strongly associated with pCR and prognosis than TILs in early HER2^+^ breast cancer [36]. This shows that immune signatures offer valuable insights with potential for predicting treatment responses.

#### ER^+^ breast cancer

ER^+^ tumors exhibit low frequency of TILs. Interestingly, the prognostic impact of TILs is not found to be favorable in this subtype. High TIL infiltration shows adverse prognosis and a shorter overall survival in a neoadjuvant therapy setting [13, 25, 28]. High Treg abundance is linked to lower ER expression [28]. Surprisingly, a high presence of Tregs in ER^+^ tumors is associated with a better prognosis [30]. NK cells are inversely correlated with ER expression status, and high infiltration is associated with good prognosis in ER^+^ breast cancers [24].

### Tumor infiltrating myeloid cells in breast cancer

Dentritic cells (DCs) are specialized antigen-presenting cells (APC) bridging the innate and adaptive immune responses. There are two distinct types of DCs: plasmacytoid DCs (pDCs) and myeloid DCs (mDCs). pDCs recognize viral infections and produce high levels of interferon type I, whereas mDCs capture, process, and present antigens to T cells [37, 38]. Circulating DCs are more prevalent in breast cancer patients compared to healthy controls [39]. The HER2^+^ subtype shows the highest amount of circulating pDCs, whereas ER^+^ subtypes have more circulating mDCs than ER^-^ subtypes [39]. Lower levels of circulating pDCs are found in patients with later stages of breast cancer [40]. Interestingly, while the presence of circulating pDC is associated with better prognosis, the infiltration into the tumor correlates with adverse outcomes [41]. TNBC exhibits high abundance of both intra-tumor and stromal immature pDC, while ER^+^ and ER^+^/HER2^+^ tumors are dominated by functional mature DCs [42]. Although DCs play a crucial role as anti-tumor cells, the tumor can induce a pro-tumorigenic DC phenotype, leading to dysfunctional and poorly activated DCs [37, 43].

Myeloid-derived suppressor cells (MDSCs) are immature myeloid cells with immune regulatory and suppressive functions [32, 44–46]. Recent studies have demonstrated that the release of cytokines, including G-CSF, IL-6, and TGF-β by breast cancer cells influences the expansion and activation of MDSCs, establishing a link between MDSCs and breast cancer progression [45]. An increased abundance of MDSCs is found in TNBC tumors [47]. In TNBC, tumor cells expressing the regulating factor ΔNp63 secrete the chemokines CXCL2 and CCL22, shown to attract MDSCs [47]. Elevated levels of MDSCs in the tumor microenvironment and in circulation are strongly associated with tumor progression and worse overall survival [46]. Furthermore, the level of circulating MDCS is higher in metastatic cancer than non-metastatic cancer [48].

Macrophages are terminally differentiated myeloid cells that can be divided into two categories with opposing actions in the TIME: pro-inflammatory M1 and immunosuppressive M2 tumor associated macrophages (TAMs) [28, 29]. The immunosuppressive M2 TAMs are the most abundant in breast cancer [49], and a high presence is associated with higher tumor grade, ER and PR negativity, and a shorter overall survival, especially in HER2^+^ and TNBC [50–52].

**Figure 2:**
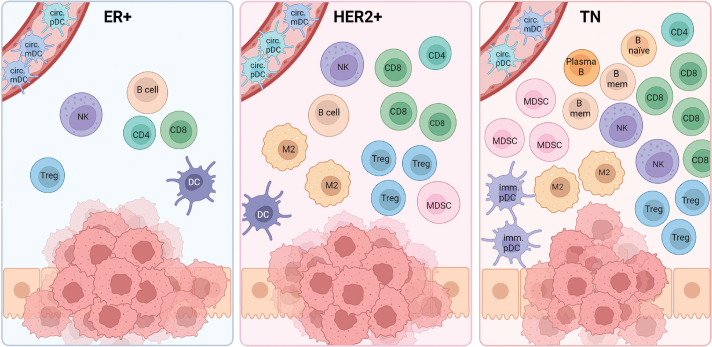
The presence of different immune cells in the molecular subtypes of breast cancer. CD8=CD8 T cell; CD4=CD4 T cell; Treg=Regulatory T cell; DC= Dendritic cell (mature); circ. mDC=Circulating myeloid DC; circ. pDC=Circulating plasmacytoid DC; imm. pDC=Immature pDC; Plasma B=Plasma B cell; B naïve=Naïve B cell; TAM=Tumor-associated macrophage; NK=Natural killer cell; MDSC=Myeloid-derived suppressor cell. Created with BioRender.com.

The precise function and composition of the different immune cells within the different breast cancer subtypes remain unclear. This underscores the challenges in interpreting the roles and functions of the cells in the microenvironment, given the highly heterogeneous nature concerning maturation and differentiation steps. The need for further investigation is evident to unravel the complexities surrounding tumor infiltrating lymphocytes and myeloid cells in breast cancer. A simplified summary of the immune composition across the molecular subtypes is given in Figure 2, and the presence and prognostic role of the different cell types are summarized in Table 2.

**Table 2:** Summary of the presence and prognostic role of the immune cells in the tumor immune microenvironment across the different subtypes of breast cancer.

Presence and prognosis of immune cells	ER^+^	HER2^+^	TNBC
Immune cell types with increased presence in breast cancer	Low immune cell infiltration, circ. mDC	TIL, CD8^+^ T cell, Treg, NK, M2 TAM, circ. pDC	TIL, CD8^+^ T cell, CD4^+^ T cell, Memory B cell, Treg, NK, imm. t-pDC, MDSC, M2 TAM
Immune cell types associated with good prognosis	B cell, Treg, circ. mDC, NK	TIL, CD8^+^ T cell, B cell, NK, circ. pDC	TIL, CD8^+^ T cell, B cell
Immune cell types associated with poor prognosis	TIL	Treg, M2 TAM	Treg, NK, imm. t-pDC, MDSC, M2 TAM

TIL=tumor infiltrating lymphocytes; NK=natural killer cells; Tregs=regulatory T cells; circ.mDC=circulating myeloid dendritic cells; circ. pDC=circulating plasmacytoid dendritic cells; imm. t-pDC=Immature tumor-infiltrating pDC; MDSCs=myeloid-derived suppressor cells.

## Tumor immune microenvironment in metastatic breast cancer

Many cancer types metastasize to predefined locations in the body, indicating that the spread is not random [53]. The hypothesis of ‘seed and soil’ was introduced by Paget over a century ago [54], where he proposed that cancer cells (seeds) are thought to thrive and grow in distant sites with favorable conditions (soil), and then ensuring their survival by altering the metastatic environment. The formation of a pre-metastatic niche is created by the primary tumor through several mechanisms including immunosuppression, inflammation, angiogenesis or vascular permeability, lymphangiogenesis, organotropism, and reprogramming [55].

### Regional metastasis

Sentinel and the axillary lymph nodes are the lymph nodes located closest to the primary tumor and serve as primary drainage for the breast tissue. Interestingly, the sentinel lymph node, and not the primary tumor, has been suggested to be the first site of tumor–immune interaction [56]. These lymph nodes are the most common sites for metastasis, and approximately 20% of breast cancer patients in Norway have spread to sentinel and regional lymph nodes at the time of diagnosis [57]. Metastatic lymph nodes display a decreased CD4^+^ to CD8^+^ T cell ratio [58, 59] and reduced frequency of DCs [59]. Furthermore, various indicators of immunosuppressive environment are noted, including elevated levels of Tregs, MDSCs, and M2 macrophages [60–62]. In metastatic lymph nodes, T cells are discovered to express cytotoxic T-lymphocyte associated protein 4 (CTLA-4), programmed death receptor 1 (PD-1), and T cell immune receptor with Ig and ITIM domains (TIGIT) and exhibit exhaustion by suppressed TCR signaling [58, 61]

### Distant metastasis

Distant metastasis involves tumor cells leaving the primary site and settling in distant organs. While early stage breast cancer has an estimated 5-year survival rate of approximately 95% and regional metastasis of 75%, the survival rate drops drastically to 27% for patients with distant metastasis [63]. The bone is the most frequent site for distant breast cancer metastasis for all subtypes, in particular for ER^+^/HER2^-^ breast cancer, and about 70% of patients with metastatic disease develop bone metastases [64, 65]. The lung and liver are the second most common site of breast cancer metastasis, followed by the brain [66, 67]. The TIME of breast cancer metastasis is highly dependent on the location of the metastasis. By measuring TIL infiltration in secondary lesions from 94 breast cancer patients, Dieci et al. [68] found that TIL levels are generally low (below 5%) in metastatic lesions. In contrast, lung metastases had a median TIL level of approximately 30%.

#### Bone

Breast cancer is likely predisposed to metastasize to the bone due to the well-vascularized nature of the bone marrow. This quality creates a nutrient-rich environment abundant in growth factors and cytokines [69]. By residing in niches in the bone marrow, tumor cells can stay dormant for decades [70]. In this environment, breast cancer cells can interact with mesenchymal stem cells (MSCs), leading to an increased production of Th2 cytokines, recruitment of Tregs, and secretion of MSC-mediated TGF-β1 [71]. These immune modulatory factors contribute to the creation of an immunosuppressive environment, allowing the cancer cells to evade immune detection and elimination by the immune system. Compared to breast lesions, bone marrow metastases show fewer macrophages and an enrichment of neutrophils, indicating an impaired antigen presentation and increased tumor-promoting cytokine secretion [72].

#### Lung

TNBC commonly metastasize to the lungs [64, 67]. While interacting with the lung stroma, the cancer cells secrete exosomes that stimulates host fibroblasts to create a pre-metastatic microenvironment, and recruit circulating monocytes that differentiate into pro-tumor macrophages [73]. This results in systemic inflammatory cascades leading to neutrophil-mediated promotion of breast cancer metastasis [74].

#### Liver

HER2^+^ breast cancer tends to metastasize to the liver [64]. While the liver is rich in immunoreactive cells, it also possesses an immunotolerant microenvironment [75]. Colonization in the liver is facilitated by the secretion of pro-inflammatory cytokines by breast cancer cells, in addition to modulating hepatocytes to increase metastasis [73]. Resident Kupffer cells, liver-specific macrophages, play a role in promoting metastasis by secreting growth factors and recruiting immunosuppressive cells like neutrophils, macrophages, and MDSCs after extravasation [76].

#### Brain

Both HER2^+^ and TNBC metastasize to the brain [77–80]. The brain and central nervous system are immune-privileged sites and are partly separated from the immune system by the blood–brain barrier. The predominant immune cell type in the brain is microglia, capable of differentiating into macrophages. The TIME in brain metastasis is identified as immunosuppressive compared to the primary breast tumor [81], with a decrease in CD8^+^ T cells and M1 macrophages, and minimal presence of B cells [82, 83]. Conversely, M2 macrophages show an opposite trend [81, 83].

## Immune checkpoint inhibitors

Immune checkpoints are regulatory pathways in the immune system, and represent important immunotherapeutic targets. Clinical trials on immunotherapy in breast cancer have increased rapidly after the discovery of immune checkpoint inhibitors, and PD1 and its ligand Programmed death receptor ligand 1 (PD-L1) are currently the most studied targets [84]. The interaction between PD-1, present on T cells, and PD-L1 and PD-L2, expressed by APCs and tumor cells, inhibits the cytotoxic effect of the immune cells, promotes T effector cell exhaustion, and promotes the conversion of T effector cells to Tregs [85]. PD-L1 inhibitors in combination with chemotherapy have demonstrated improved progression-free survival for both PD-L1^+^ (KEYNOTE-355 [86], KEYNOTE-522 [87], IMPASSION130 [88]) and PD-L1^-^ patients (ALICE [89]). The PD-L1 inhibitor pembrolizumab, in combination with chemotherapy, is approved and used as standard of care in several countries in the treatment of metastatic PD-L1^+^ TNBC [90–92]. Targeting other immune checkpoint molecules such as T-cell immunoglobulin and mucin domain 3 (TIM-3), TIGIT and Lymphocyte-activation gene 3 (LAG-3) could potentially offer additional novel therapies. Tregs express immune checkpoints, and may be an unintended target for immune checkpoint inhibitors. Blomberg et al. recently discovered that depletion of Tregs in combination with adjuvant checkpoint inhibitors prolonged metastasis-related survival in breast cancer in mice, thus indicating that this could be a potential empowerment of checkpoint therapy [93].

## Concluding remarks

The complexity of the tumor infiltrating lymphocytes and myeloid cells, comprising various immune cells, necessitates a deeper exploration of their interplay within the TIME. Technological advancements like single-cell sequencing and multiplexing offer opportunities for more comprehensive analyses, elucidating the dual role of immune cells as both anti-tumor and pro-tumor entities and the interplay between the different cell types. However, numerous aspects remain unknown, emphasizing the need to contextualize immune cell interactions within specific breast cancer subtypes and in various metastatic sites. Integrating emerging technologies and gaining deeper understanding of various immune cell types in breast cancer microenvironment are pivotal for unraveling complexities, refining prognostic and therapeutic strategies tailored to each subtype.

## Data Availability

Data sharing is not applicable as no new data generated.
